# Evaluating tuberculosis treatment outcomes in Haiti from 2018 to 2019: A competing risk analysis

**DOI:** 10.1016/j.ijregi.2024.03.005

**Published:** 2024-03-13

**Authors:** Nernst-Atwood Raphael, Pierre Anthony Garraud, Maroussia Roelens, Jean Patrick Alfred, Milo Richard, Janne Estill, Olivia Keiser, Aziza Merzouki

**Affiliations:** 1 Institute of Global Health, University of Geneva, Geneva, Switzerland; 2 Strategic Health Information System, DAI Global LLC, Port-au-Prince, Haiti; 3 Unit of Studies and Programming, Ministry of Health, Port-au-Prince, Haiti; 4 National Tuberculosis Program, Ministry of Health, Port-au-Prince, Haiti

**Keywords:** Competing risk, TB treatment outcomes, Lower- and middle-income countries, Treatment duration and demographics, Improved tuberculosis management

## Abstract

**Objectives:**

This study assesses tuberculosis (TB) treatment outcomes in Haiti.

**Methods:**

Data from drug-susceptible patients with TB (2018-2019) were analyzed using the Fine & Gray model with multiple imputation.

**Results:**

Of the 16,545 patients, 14.7% had concurrent HIV coinfection, with a 66.2% success rate. The median treatment duration was 5 months, with patients averaging 30 years (with an interquartile range of 22-42 years). The estimated hazard of achieving a successful treatment outcome decreased by 2.5% and 8.1% for patients aged 45 and 60 years, respectively, compared with patients aged 30 years. Male patients had a 6.5% lower estimated hazard of success than their female counterparts. In addition, patients coinfected with HIV experienced a 35.3% reduction in the estimated hazard of achieving a successful treatment outcome compared with those with a negative HIV serologic status.

**Conclusions:**

Integrated health care approaches should be implemented, incorporating innovative solutions, such as machine learning algorithms combined with geographic information systems and non-conventional data sources (including social media), to identify TB hotspots and high-burden households.

## Highlights

• Age, gender, HIV, tuberculosis form, location, urbanity, and facility affected outcomes.
• Coinfected patients faced separate clinics, hindering collaboration and outcomes.
• Suggest using community case-finding and machine learning to detect and treat missed tuberculosis cases.
• Recommendations for integrated care and digital medication reminders.
• Suggest using digital medication reminders to prevent patient loss to follow-up.

## Introduction

Before 2020, tuberculosis (TB) was the leading cause of death from a single infectious agent, ranking above HIV/AIDS [1]. Nowadays, TB continues to be a public health threat and, in many places, it has been aggravated by the pandemic [1]. In 2021, 10.6 million TB cases were diagnosed worldwide, killing more than 1.6 million persons [1]. The true number of cases is believed to be higher than reported owing to the disruptions of health care caused by COVID-19 during the past years [1]. The World Health Organization (WHO) is supporting the National Tuberculosis Program from 194 member states to end the pandemic. In 2015, several international and philanthropic organizations, in addition to other stakeholders, joined in a collective effort to end TB by 2035 [2].

In 2022, Haiti reported 11,433 TB cases, constituting 63.0% of the true TB cases nationwide. About 90% were receiving TB treatment for the first time. The West, Artibonite, and North departments comprised over half (56.5%) of the cases. Approximately 1595 patients with TB were coinfected with HIV. The national treatment success rate was estimated at 81.6% for the 2021 cohort [3].

Several strategies were put in place to increase the TB treatment efficacy [4]. In this vein, numerous randomized clinical trials have been conducted since the discovery of the first-line TB treatment to assess the safety and efficacy of a shorter treatment. In a recently published study, the authors provided sufficient evidence for noninferiority of a 4-month treatment to the standard 6-month regimen [5]. Nonetheless, additional obstacles, including poor adherence to the treatment, impede the end of TB. These obstacles need to be addressed. Poor adherence has been linked to diagnostic procedures; hospitality of health personnels [6,7]; discrimination and stigmatization against patients [6], [7], [8]; poor knowledge of TB, modes of transmission, and duration of treatment [6,7]; long distance to health care facilities [7]; therapeutic adverse effects [7,8]; and socioeconomic factors such as food scarcity [6], [7], [8], [9].

Poor adherence to the treatment is linked with unfavorable treatment outcomes (death, loss to follow-up, or treatment failure) [10], [11], [12]. Little is known regarding the determinants of unfavorable outcomes of patients with TB in the Haitian context. This study aimed to assess the competing risks of a successful treatment outcome of patients receiving TB care in Haiti. A secondary aim is the provision of information that may help guide policymakers in the implementation of the TB program in Haiti and transferable knowledge for other lower- and middle-income countries. Furthermore, the study may uncover patterns or trends in TB treatment outcomes that could be indicative of broader issues within the health care system. For example, if certain regions consistently show poorer treatment outcomes, this could prompt further investigation into potential barriers to accessing care, variations in health care quality, or other systemic issues that need to be addressed.

## Materials and methods

We conducted a longitudinal analysis of patients enrolled in the TB program implemented by the National Tuberculosis Program in Haiti (PNLT) between 2018 and 2019. All patients with TB were treated using the WHO-recommended protocol of a 6-month treatment consisting of 2 months of isoniazid (H), rifampicin (R), pyrazinamide (Z), and ethambutol (E), followed by 4 months of isoniazid (H) and pyrazinamide (Z): 2HRZE/4HR [13]. The United States Agency for International Development funded the Development Alternatives Incorporated to implement the Haiti Strategic Health Information System (HIS) to support the Ministry of Health of Haiti (MSPP) to sustain and enhance Haiti's national HIS. Using digital health solutions (e.g. District Health Information System 2 [DHIS2] tracker), HIS developed a TB tracker software to improve the follow-up of patients. The tracker has four modules which encompass patient-level data on drug-susceptible TB, multidrug-resistant TB, and isoniazid prophylaxis, respectively, and contact tracing of patients with bacteriologically confirmed pulmonary TB. Between January and February 2018, the PNLT and the Unit of Studies and Programming of the MSPP, with the technical and financial support of HIS, piloted the TB tracker in selected health facilities before its national deployment on March 1, 2018.

The data set under study consisted of patients enrolled in the national drug-susceptible TB and patient care program. The information system follows the same process as suggested by the WHO for implementing any national TB program [13]. Patients with pulmonary TB were either bacteriologically confirmed (smear +, detected by Gene Xpert or culture) at the beginning of treatment or clinically confirmed. In the case of extrapulmonary TB, the patient diagnosis was conducted as a combination of chest X-ray and other relevant diagnostic tests based on the symptoms. Between January 1, 2018, and March 31, 2018, the HIS extensively implemented a pilot of the TB tracker. The TB tracker was developed in DHIS2 and, at this preliminary version, consisted of the drug-susceptible module. Upon successful completion of the pilot phase, the national rollout of the TB tracker was effective on April 1, 2018. The authors believe that using the official national rollout date as the starting date of the cohort would improve data quality because all health care facilities providing TB care implemented the same system.

Patients enrolled in the TB program from April 1, 2018, that is, when the TB tracker was started to be implemented throughout the country, were eligible to enter this study. Starting in July 2019, owing to the highly volatile political situation in the country, including roadblocks, a sharp increase in kidnappings, and countrywide lockdowns, physical movements to the health facilities became challenging, making technical onsite support more difficult. Because the unstable situation caused serious limitations for data collection and quality, we decided to end the study on June 30, 2019. The exclusion criteria were patients with missing treatment initiation date, exit date (defined as the date at which the outcome occurred or was recorded), or diagnostic date and those whose diagnosis date was later than their initiation date.

The variable of interest was the outcome of the TB treatment. The covariates included (as shown in Table 1, Table 2): age of the patients (continuous), gender (female or male), residence setting (urban or rural), department/district (Artibonite, Central Plateau, Grand-Anse [southwest]), Nippes, North, Northeast, Northwest, South, Southeast, and West), TB form (pulmonary or extrapulmonary), type of treatment buddy (friend, community member, patient, or parent), HIV coinfection (yes or no), health facility type (public, private, or mixed), and treatment history (new or retreatment). The health facilities settings were classified as urban or rural. Other subcategorization included departments (the 10 geographical and administrative regions of Haiti). Using funding mechanisms, we categorized the health facilities as public, private, or mixed. We computed the duration of the treatment as the difference between the exit date and the date of treatment initiation in the program in days. Patients who were still on treatment after the study end date were right censored. Proportions, medians, and interquartile range were used to present descriptive statistics. Chi-squared tests were computed to compare the proportions between groups.

**Table 1:** Characteristics of the population under study.

Department	Frequency	Percent
Artibonite	2,325	14.1
Centre	1,121	6.8
Southwest (Grand'Anse)	873	5.3
Nippes	549	3.3
North	1,325	8.2
Northeast	713	4.3
Northwest	877	5.3
West	6,860	41.4
South	1,123	6.8
Southeast	749	4.5
Gender		
Female	7,291	44.1
Male	9,254	55.9
Type of treatment buddy		
Parent	14,006	84.7
Other	1,301	7.8
Missing	1,238	7.5
Tuberculosis form		
Extrapulmonary	1,712	10.3
Pulmonary	14,789	89.4
Missing	44	0.3
Treatment history		
New	15,011	90.7
Retreatment	1,482	9.0
Missing	52	0.3
Treatment outcome		
Death	886	5.4
Treatment failure	105	0.6
Favorable (Cured, or treatment completed)	11,121	67.2
Loss to follow up	1,397	8.4
Censored (Still in care, or not evaluated)	3,036	18.4
HIV status		
Negative	13,396	81.0
Positive	2,424	14.7
Missing	725	4.3
Residence setting		
Urban	9.575	57.9
Rural	6.970	42.1
Health facility type		
Public	7,068	42.7
Private	7,412	44.8
Mixed	2,065	12.5
Total	16,545	100.0

**Table 2:** Characteristics of the population under study.

	Mean (Standard deviation)	Median (Interquartile range)
Age (in years)	32.5 (15.6)	30 (22-42)
Duration of treatment (in months)	5.3 (1.7)	6 (5-6)

The survival analysis was conducted using the competing risks Fine & Gray model, with age included as restricted cubic splines using 35 as the reference category and 20, 45, and 60 years as cut-off ages [14], [15], [16], [17], [18], [19]. The outcome of interest was treatment success defined as the patient being either cured, or having completed the treatment without failure. Death, loss to follow-up, and treatment failure were competing events. Data from patients still in care and those not evaluated (mostly because of transfer) were censored. Cumulative incidence functions were produced for all treatment outcomes (as described in Box 1, Supplementary Material). The regressions on the chained equations have been iterated 10 times using the entire set of variables. A sensitivity analysis was conducted comparing two versions of the model with and without multiple imputation by chained equations. Subdistribution hazard ratios alongside 95% confidence intervals were produced. Data management and analysis were performed using R 3.6.1 [20,21]. Ethics approval, reference number 1921-2, was obtained from the National Bioethics Committee of MSPP for the analysis of the data in the context of this study. Because this study used secondary data from patient medical records, no approval was necessary from the institutional review board office at the University of Geneva. A de-identified replicate of the data set was downloaded from DHIS2 TB tracker and exported to R [20] for data management and analysis. The data set was password protected and its access has been granted solely to the principal investigator and study collaborators from the University of Geneva. Table 2.

## Results

A total of 16,545 patients were included in the analysis (as shown in Table 1), with the West department (including the metropolitan area of the Capital, Port-au-Prince) accounting for 41.5% of the patients, followed by Artibonite with 14.1%. Patients were predominantly male (55.9%), with a member of their family as a treatment buddy in 84.7% of the cases. Nine of 10 (89.4%) patients had a pulmonary form of TB; for 90.7% of the patients, they were treated for the first time. HIV coinfection was present in 14.7% of the patients. Two-thirds (66.2%) of the patients had a successful outcome. The median treatment duration was 5 (interquartile range 1-6) months (as shown in Table 2). The median age among patients was 30 (interquartile range 22-42) years. Overall, 42.1% of the health facilities were in rural settings and 42.7% were public facilities.

The bivariate analysis of TB treatment outcome is displayed in Table 3. The proportion of patients with a successful treatment outcome among women was 67.3% compared with 65.9% among men. There was a statistically significant (*P* <0.001) association between treatment outcomes and gender. The proportion of patients with a successful treatment outcome among those with pulmonary TB was 66.4% compared with 67.2% among those with extrapulmonary TB. There was a statistically significant (*P* <0.001) relationship between treatment outcomes and TB form. Patients who initiated a TB treatment for the first time had a 66.9% chance of a successful treatment outcome compared with 63.3% for those who were retreated. The association between treatment outcome and treatment history was statistically significant (*P* <0.001). The proportion of patients with a successful treatment outcome among those with an HIV-negative serologic status was 69.0% compared with 56.3% among those coinfected with HIV. There was a statistically significant (*P* <0.001) relationship between treatment outcomes and HIV serologic status.

**Table 3:** Repartition of treatment outcomes per key factors.

	Key factors		Treatment outcome (%)					Chi-squared *P*-value
		Total	Censored	Success	Death	Loss to follow up	Treatment failure	
Gender	Female	7,291	1,376 (18.9)	4,962 (68.1)	353 (4.8)	43 (0.6)	557 (7.6)	<0.05
	Male	9,254	1,660 (17.9)	6,159 (66.5)	533 (5.8)	62 (0.7)	840 (9.1)	<0.05
	Total	16,545	3,036 (18.4)	11,121 (67.2)	886 (5.4)	105 (0.6)	1,397 (8.4)	<0.05
Treatment buddy type	Other	1,301	250 (19.2)	866 (66.6)	81 (6.2)	4 (0.3)	101 (7.7)	<0.05
	Parent	14,006	2,568 (18.3)	9,522 (68.0)	741 (5.3)	93 (0.7)	1,082 (7.7)	<0.05
	Total	15,307	2,818 (18.4)	10,388 (67.9)	822 (5.4)	97 (0.6)	1,182 (7.7)	<0.05
Tuberculosis form	Extrapulmonary	1,712	251 (14.7)	1,177 (68.7)	138 (8.1)	2 (0.1)	144 (8.4)	<0.05
	Pulmonary	14,789	2,778 (18.8)	9,915 (67.1)	744 (5.0)	103 (0.7)	1,249 (8.4)	<0.05
	Total	16,501	3,029 (18.4)	11,092 (67.2)	882 (5.4)	105 (0.6)	1,393 (8.4)	<0.05
Treatment history	Retreatment	1,482	264 (17.8)	952 (64.2)	87 (5.9)	22 (1.5)	157 (10.6)	<0.05
	New treatment	15,011	2,762 (18.4)	10,139 (67.5)	791 (5.3)	83 (0.6)	1,236 (8.2)	<0.05
	Total	16,493	3,026 (18.4)	11,091 (67.3)	878 (5.3)	105 (0.6)	1,393 (8.4)	<0.05
HIV status	Negative	13,396	2,494 (18.3)	9,337 (69.7)	476 (3.6)	80 (0.6)	1,009 (7.5)	<0.05
	Positive	2,424	425 (17.5)	1,376 (56.8)	341 (14.1)	20 (0.8)	262 (10.8)	<0.05
	Total	15,820	2,919 (18.5)	10,713 (67.7)	817 (5.2)	100 (0.6)	1,271 (8.0)	<0.05
Department/District	West	6,860	1,234 (18.0)	4,569 (66.6)	322 (4.7)	35 (0.5)	700 (10.2)	<0.05
	Southeast	749	124 (16.6)	528 (70.5)	35 (4.7)	5 (0.7)	57 (7.6)	<0.05
	North	1,355	207 (15.3)	942 (69.5)	114 (8.4)	15 (1.1)	77 (5.7)	<0.05
	Northeast	713	137 (19.2)	494 (69.3)	44 (6.2)	0 (0.0)	38 (5.3)	<0.05
	Artibonite	2,325	487 (20.9)	1,554 (66.8)	131 (5.6)	14 (0.6)	139 (6.0)	<0.05
	Centre	1,121	233 (20.8)	743 (66.3)	31 (2.8)	13 (1.2)	101 (9.0)	<0.05
	South	1,123	203 (18.1)	770 (68.6)	63 (5.6)	10 (0.9)	77 (6.9)	<0.05
	Southwest	873	186 (21.3)	565 (64.7)	43 (4.9)	4 (0.5)	75 (8.6)	<0.05
	Northwest	877	128 (14.6)	573 (65.3)	63 (7.2)	8 (0.9)	105 (12.0)	<0.05
	Nippes	549	97 (17.7)	383 (69.8)	40 (7.3)	1 (0.2)	28 (5.1)	<0.05
	Total	16,545	3,036 (18.3)	11,121 (67.2)	886 (5.4)	105 (0.6)	1,397 (8.4)	<0.05
Urban setting	Urban	9,575	1,687 (17.6)	6,452 (67.4)	510 (5.3)	57 (0.6)	869 (9.1)	<0.05
	Rural	6,970	1,349 (19.4)	4,669 (67.0)	376 (5.4)	48 (0.7)	528 (7.6)	<0.05
	Total	16,545	3,036 (18.3)	11,121 (67.2)	886 (5.4)	105 (0.6)	1,397 (8.4)	<0.05
Health facility type	Public	7,068	1,342 (19.0)	4,856 (68.7)	365 (5.2)	37 (0.5)	468 (6.6)	<0.05
	Private	7,412	1,295 (17.5)	4,909 (66.2)	415 (5.6)	53 (0.7)	740 (10.0)	<0.05
	Mixed	2,065	399 (19.3)	1,356 (65.7)	106 (5.1)	15 (0.7)	189 (9.2)	<0.05
	Total	16,545	3,036 (18.3)	11,121 (67.2)	886 (5.4)	105 (0.6)	1,397 (8.4)	<0.05

**Figure 1:**
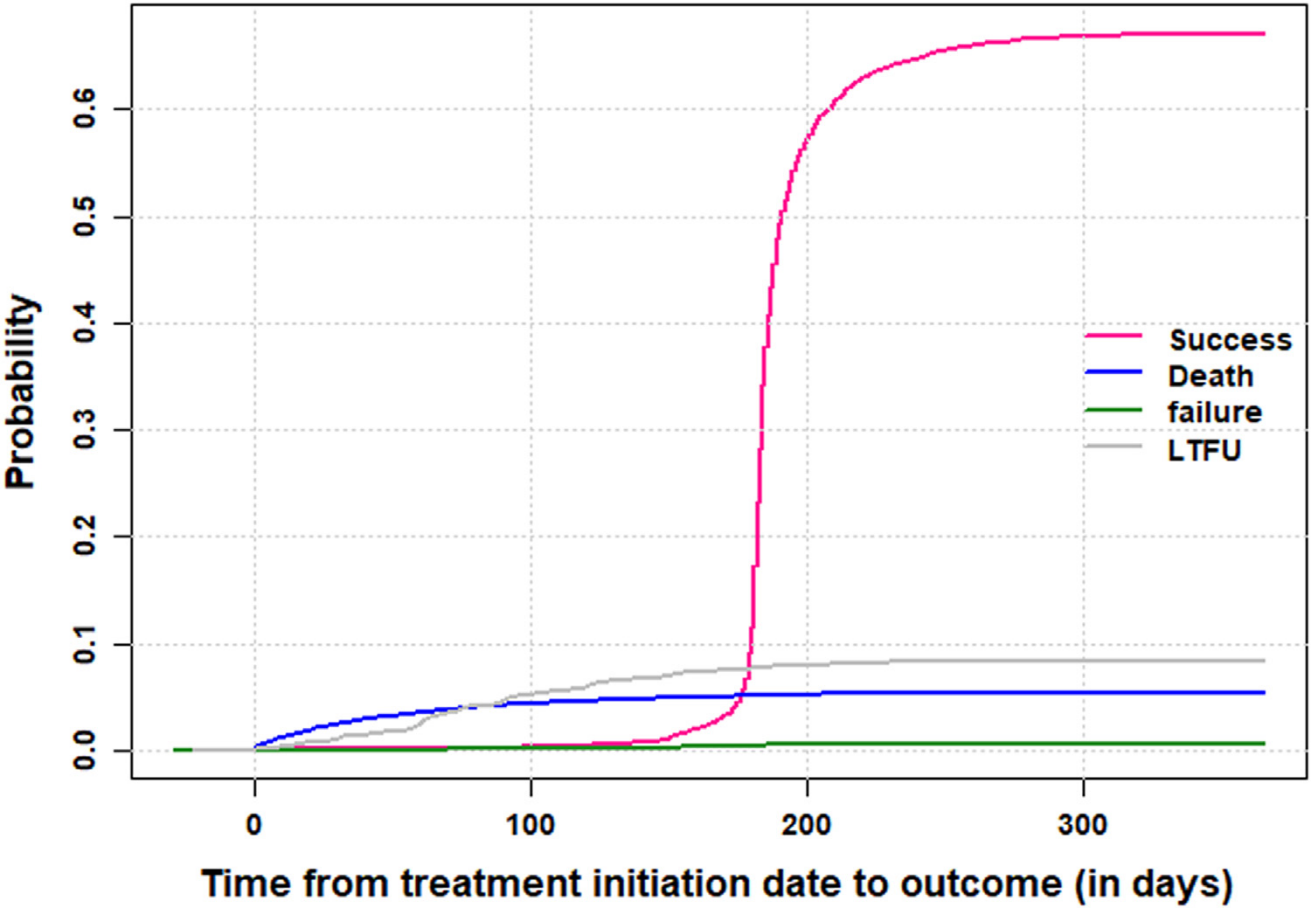
Cumulative indcidence functions. LTFU, loss to follow-up.

The cumulative incidence function of TB treatment outcome (as shown in Figure 1) is growing constantly to 62.0% after the first 6 months (because of the minimum duration of treatment ∼ 180 days).

The adjusted competing risk analysis using multiple imputation by chained equations is displayed in Table 4. The estimated hazard to have a successful outcome (either cured or completed treatment) for a patient with TB aged 45 years (60 years, respectively) decreased by 2.3% (7.1%, respectively) compared with a patient aged 35 years, controlling for gender, HIV status, treatment buddy type, TB type, the administrative location in which the patient has been treated, urbanity of the setting, and health facility type.

**Table 4:** Results of the competing risks models using Fine & Gray.

Covariates	Adjusted without imputation				Adjusted with mice^a^			
	Sub. HR^b^	Sub. HR 95% CI	*P*-value	Sub. HR^c^	Sub. HR 95% CI	*P*-value		
Age = 35	1.000	1.000	1.000	0.025	1.000	1.000	1.000	0.002
Age = 20	0.999	0.972	1.027		1.006	0.979	1.033	
Age = 45	0.981	0.967	0.995		0.977	0.965	0.990	
Age = 60	0.937	0.894	0.983		0.929	0.888	0.971	
Gender: Female	1.000	1.000	1.000	0.153	1.000	1.000	1.000	0.022
Gender: Male	0.971	0.934	1.011		0.957	0.922	0.994	
HIV status: Negative	1.000	1.000	1.000	<0.001	1.000	1.000	1.000	<0.001
HIV status: Positive	0.619	0.583	0.658		0.624	0.588	0.661	
Type of treatment buddy: Other	1.000	1.000	1.000	0.319	1.000	1.000	1.000	0.299
Type of treatment buddy: Parent	0.959	0.885	1.0417		0.959	0.885	1.038	
Tuberculosis type: Extrapulmonary	1.000	1.000	1.000	0.094	1.000	1.000	1.000	0.464
Tuberculosis type: Pulmonary	0.946	0.887	1.009		0.939	0.884	0.999	
Department: West	1.000	1.000	1.000	<0.001	1.000	1.000	1.000	<0.001
Department: Southeast	1.009	0.906	1.124		1.033	0.935	1.141	
Department: North	0.881	0.815	0.952		0.921	0.854	0.993	
Department: Northeast	1.234	1.110	1.372		1.310	1.183	1.452	
Department: Artibonite	0.982	0.915	1.054		1.059	0.989	1.134	
Department: Centre	1.104	0.981	1.242		1.240	1.117	1.377	
Department: South	0.884	0.812	0.962		0.947	0.873	1.027	
Department: Southwest	0.893	0.810	0.984		0.957	0.869	1.052	
Department: Northwest	0.939	0.856	1.029		1.026	0.939	1.121	
Department: Nippes	1.034	0.924	1.158		1.086	0.972	1.214	
Residence setting: Urbaniii	1.000	1.000	1.000	0.097	1.000	1.000	1.000	
Residence setting: Rural	1.018	0.967	1.071		0.989	0.944	1.038	0.673
Health facility type: Publiciii	1.000	1.000	1.000	0.003	1.000	1.000	1.000	<.001
Health facility type: Private	0.946	0.905	0.989		0.920	0.881	0.961	
Health facility type: Mixed	0.881	0.807	0.964		0.885	0.813	0.963	

CI, confidence interval; HR, hazard ratio; Sub. HR, subdistribution HR.

a Multiple imputation by chained equations.

iii Indicates that the label serves as the reference category.

There was a 4.3% decrease in the estimated hazard of a successful outcome for a male patient compared with a female patient with TB. The estimated hazard of a successful outcome for a patient with TB coinfected with HIV decreased by 37.6% compared with a patient with TB with a negative HIV serologic status.

The estimated hazard of a successful outcome for patients with TB treated in a health facility in the North experienced a 7.9% decrease compared with those who received care in a health facility located in the West department. The estimated hazard of a successful outcome for patients with TB treated in a health facility in the Northeast (the Center, respectively) increased by 31.0% (24.0%, respectively) compared with those who received care in a health facility located in the West department. The estimated hazard to have a successful outcome for a patient with TB receiving care in a private (mixed, respectively) health facility setting decreased by 8.0% (11.5%, respectively) compared with patients treated in a health facility with a public setting.

## Discussion

The longitudinal data of patients receiving care for TB enrolled in a surveillance program under the PNLT between 2017 and 2019 in Haiti were analyzed. Consistent with the findings of other studies, HIV-positive status was linked to poorer treatment outcomes [22], [23], [24], [25]. In addition to the potential psychological effects of having to take several (HIV plus TB) pills, there are other arguments for this association. For instance, in Haiti, patients with TB coinfected with HIV attend two different clinics (one for TB and one for HIV) within the same health facility, with only the penitentiary clinics providing integrated care. The funds allocated to HIV clinics, including human resources and salary among staff, are substantially higher than those in TB clinics, which creates a barrier for effective collaboration between the two clinics. As a result, there is a high likelihood that the follow-up visits for a patient with TB coinfected with HIV differ in these clinics, leading to an increased risk of poor treatment outcomes.

Male patients with TB have a significantly lower chance of a successful outcome [24]. Although it is difficult to estimate the magnitude of the problem, during data quality workshops, some health care providers reported instances where male patients sell their food portions provided to them by the program instead of consuming them or used their transportation fees for other purposes, which may contribute to poor treatment outcome. The authors believe that it is safe to say that men, often seen as primary breadwinners in Haiti, may face pressures to resume income-generating activities sooner during the treatment period, potentially leading to an increased likelihood of treatment failure and loss to follow-up.

Successful treatment outcomes are found to be significantly higher in public health facilities than in private or mixed ones. Dr. Milo, from the PNLT in Haiti, suggested that the health facilities categorized as public are the ones where the turnover rates are the lowest. These facilities also have more experienced health care providers who are better equipped to deal with patients with TB. The combination of experience in dealing with patients with TB and the low turnover rates among these public health facilities seems to contribute to the higher success rate in TB treatment outcomes.

To improve the quality of care provided to patients with TB and to increase their odds of a more favorable treatment outcome, there is a need for strong political leadership at all levels. In addition, there is a need to substantially improve the integration of health services provided within a health facility. Informed decisions through continued strengthening of the HIS, the use of “Carte Sanitaire” to assess accessibility to TB care, and continued capacity building are among the key solutions to address the challenges facing TB control in Haiti. Although little evidence is available on the subject, community-based active case-finding interventions for TB may be a useful strategy in identifying and treating cases missed by conventional case detection [26]. Furthermore, using machine learning algorithms combining geographic information systems and non-conventional data sources, including social media and other conventional sources risk factors, can help predict TB hotspots and/or high-burden households [27]. Adding a social protection component to the TB treatment may improve the treatment outcomes [28]. In addition, implementing a digital medication event reminder may prevent loss to follow-up [29,30].

In late January of 2023, the PNLT convened a workshop to update their TB treatment guidelines to include the new WHO recommendations for the 4-month regimen of isoniazid, rifapentine, moxifloxacin, and pyrazinamide [11]. A pilot of the 4-month regimen of isoniazid, rifapentine, moxifloxacin, and pyrazinamide will be implemented at selected health facilities. In this study, all patients received the same 6-month treatment duration. The authors hypothesize that reducing the treatment duration will positively impact treatment outcomes. One of the reasons for poor adherence is the long duration of the course, and shortening the treatment can improve adherence and thus treatment outcomes.

### Strengths and limitations

Our study offers a comprehensive analysis of longitudinal data on patients with TB in Haiti, encompassing various factors influencing treatment outcomes. This thorough examination provides a robust foundation for understanding the complexities of TB care in the country.

However, despite its strengths, our study is not without limitations. Notable weaknesses include the absence of data on certain factors, such as comorbidities, type of health care provider, history of bacille Calmette-Guerin vaccination, wealth index, education level, distance to the health facility, behavioral factors (smoking and knowledge of the disease), waiting time, food accessibility, and food consumption, which could have provided additional insights into treatment outcomes.

## Conclusion

Integrated health care approaches should be implemented, incorporating innovative solutions, such as machine learning algorithms combined with geographic information systems and non-conventional data sources (including social media), to identify TB hotspots and high-burden households. Furthermore, the use of digital medication event reminders could help prevent loss to follow-up among patients.

By embracing these suggested interventions, with a special emphasis on the implementation of the 4-month regimen, policymakers can make significant strides in enhancing TB treatment outcomes and strengthening surveillance systems in resource-limited settings. This approach holds great potential for reducing the burden of TB and improving public health outcomes for vulnerable populations.

## Declarations of competing interest

MR and JPA are members of the Ministry of Health of Haiti, an institution that received several bilateral fundings. During the preparation of the first draft of the manuscript, NAR worked for Development Alternatives Inc., recipient of funding from the United States Agency for International Development (AID-521-A-17-00008). AM and OK worked under the professorship grant (n°196270 and n°202660) provided by the Swiss National Science Foundation (SNF). The funders had no role in the study design, data collection and analysis, decision to publish, or manuscript preparation. All other authors declare no competing interests. Furthermore, they are responsible for the content and writing of the paper.

## Appendix Supplementary materials

application 1 Image, application 1
