# Supplementary material for: Evaluating tuberculosis treatment outcomes in Haiti from 2018 to 2019: A competing risk analysis
Source: IJID Reg. 2024 Mar 13;11:100350. doi: 10.1016/j.ijregi.2024.03.005 (PMC10993134; doi:10.1016/j.ijregi.2024.03.005)
Supplement: application 1 [file mmc1.docx]

|  | Box 1: Treatment outcome - Definition |
| --- | --- |
| Favorable outcome |  |
| Cured | A pulmonary TB patient with bacteriologically confirmed TB at the beginning of treatment who was smear- or culture-negative in the last month of treatment and on at least one previous occasion. |
| Treatment completed | A TB patient who completed treatment without evidence of failure but with no record to show that sputum smear or culture results in the last month of treatment and on at least one previous occasion were negative, either because tests were not done or because results are unavailable. |
| Unfavorable outcome |  |
| Treatment failure | A TB patient whose sputum smear or culture is positive at month five or later during treatment. |
| Death | A TB patient who dies for any reason before or during treatment. |
| Loss to follow up (LTFU) | A TB patient whose treatment was interrupted for two consecutive months or more. |
| Censored |  |
| Still in care | A TB whose duration in treatment is less than 6 months for whom treatment outcome was unknown at the end of the study period. |
| Missing |  |
| Not evaluated | A TB patient transferred to another treatment facility for whom treatment outcome is unknown to the reporting facility. |
| Not reported | A TB patient whose duration of treatment exceeds 8 months for whom no treatment outcome was assigned at the end of the study period. |
